# In Vitro Investigation of the Antibacterial Activity of Nine Commercial Water Disinfectants, Acidifiers, and Glyceride Blends against the Most Important Poultry Zoonotic Bacteria

**DOI:** 10.3390/pathogens12030381

**Published:** 2023-02-27

**Authors:** Tilemachos Mantzios, Vasilios Tsiouris, Konstantinos Kiskinis, Vangelis Economou, Evanthia Petridou, Anestis Tsitsos, Apostolos Patsias, Ioanna Apostolou, Georgios A. Papadopoulos, Ilias Giannenas, Paschalis Fortomaris

**Affiliations:** 1 Unit of Avian Medicine, Clinic of Farm Animals, School of Veterinary Medicine, Aristotle University of Thessaloniki, 54627 Thessaloniki, Greece; 2 Laboratory of Food Animal Hygiene and Veterinary Public Health, School of Veterinary Medicine, Aristotle University of Thessaloniki, 54124 Thessaloniki, Greece; 3 Laboratory of Microbiology and Infectious Diseases, School of Veterinary Medicine, Aristotle University of Thessaloniki, 54124 Thessaloniki, Greece; 4 Agricultural Poultry Cooperation of Ioannina “PINDOS”, Rodotopi, 45500 Ioannina, Greece; 5 National Reference Laboratory (NRL) for Campylobacter, Veterinary Laboratory of Ioannina, 45221 Ioannina, Greece; 6 Laboratory of Animal Science, School of Veterinary Medicine, Aristotle University of Thessaloniki, 54124 Thessaloniki, Greece; 7 Laboratory of Nutrition, School of Veterinary Medicine, Aristotle University of Thessaloniki, 54124 Thessaloniki, Greece

**Keywords:** zoonotic bacteria, poultry, organic acids, glyceride blends, water disinfection, water acidification, antibacterial activity, minimal inhibitory concentration (MIC)

## Abstract

Identifying and monitoring the efficiency of alternative biocides that are presently used in livestock is gaining vast attention. The objective of this study was to determine, in vitro, the antibacterial activity of nine commercial water disinfectants, acidifiers, and glyceride blends against clinical isolates or reference strains of zoonotic pathogens belonging to the genera *Escherichia* spp., *Salmonella* spp., *Campylobacter* spp., *Listeria* spp., and *Staphylococcus* spp. For each product, the antibacterial activity was tested in concentrations ranging from 0.002 to 1.136% *v/v* and expressed as the minimum concentration of the product that inhibits bacterial growth (MIC). Water disinfectants Cid 2000™ and Aqua-clean^®^ recorded MICs ranging from 0.002 to 0.142% *v/v*, while the lowest MICs were recorded at two strains of *Campylobacter* (0.002–0.004% *v/v*). Virkon^®^ S displayed various MICs (0.013–0.409% *w/v*) and was highly effective at suppressing the growth of Gram-positive bacteria such as *S. aureus* (0.013–0.026% *w/v*). The MICs of water acidifiers (Agrocid Super™Oligo, Premium acid, and Ultimate acid) and glyceride blends (CFC Floramix, FRA^®^LAC34, and FRA^®^Gut Balance) ranged from 0.036 to 1.136% *v/v*, and for most of these products, MICs were closely correlated by their ability to modify the pH of the culture medium close to 5. In conclusion, most of the tested products showed promising antibacterial activity; as a result, they would be good candidates for pathogen control in poultry farms and for reducing the emergence of antimicrobial resistance. However, further in vivo studies are recommended to provide relevant information for the underlying mechanisms, as well as for the establishment of the optimal dosage scheme for each product and their possible synergies.

## 1. Introduction

Poultry products, particularly chicken meat and eggs, are important protein sources in the human diet. According to the Food and Agriculture Organization, poultry meat production ranked first in 2019, accounting for 39% of global meat output (130.5 million tons), while global egg production reached 82.17 million metric tons [1]. In order to fulfill the ever-increasing consumer demand, the poultry industry has become more intensive than ever and represents one of the fastest-developing segments of agriculture. However, intensive production necessitates an increase in livestock density, which is a factor that may significantly alter transmission patterns and the conditions under which various pathogens could trigger the emergence of zoonotic diseases [2].

According to the European Union One Health 2019 Zoonoses Report, *Campylobacter* spp. (the most reported zoonosis in humans), *Salmonella* spp. and Shiga toxin-producing *Escherichia coli* (STEC) are frequently isolated from poultry products available in the market, whereas *Listeria* spp. rarely exceeded the EU food safety limit [3]. From the pathogens above, *C. jejuni* is considered to be the main causative agent of human bacterial gastroenteritis [4,5], and the frequently isolated antibiotic-resistant *C. jejuni* strains, in both humans and animals, has led health organizations to embrace intervention strategies based upon the concept of “One Health”. A strong correlation between the flock infection status and carcass contamination level is frequently recorded [6,7]. Therefore, there is an urgent demand to control infections at the farm level [8].

Antibiotics have been used therapeutically and prophylactically in the poultry industry since the 1940s [9,10]. The use of antibiotics at low or subtherapeutic doses for long periods was found to promote animal growth by augmenting nutrient digestibility, adjusting the balance of intestinal microbiota, and contributing to the absorbance capacity of the intestine [11]. Moreover, antibiotic utilization permits larger stocking densities, camouflages stress-related immunosuppression, and could prevent the transmission of zoonotic pathogens [12]. However, antibiotic growth promoters (AGPs) in animal feed have been associated with increasing food-related antibiotic-resistant bacteria [13]. Gram-negative bacteria of animal origin, such as *Campylobacter* spp., *Salmonella* spp., and *Escherichia coli*, have been increasingly resistant to many antibiotics [14].

Since 1 January 2006, according to EC Regulation No. 1831/2003, the European Union has entirely banned the use of antibiotics as growth promoters in animal feed [15]. In addition, since 1 January 2017, according to Guidance for Industry (GFI) #213, the United States has completely banned the use of “Critically Important” for human medicine antibiotics as growth promoters in livestock [16]. Withdrawal of AGPs has led to nutritional disorders, poor performance, and a rise in bacterial disease incidence, such as necrotic enteritis and dysbacteriosis [17]. Therefore, there is an urgent demand for the development of alternative biocide compounds and amendments. These may partially or fully replace antibiotics in poultry and could be efficient both in reducing the incidence of bacterial infections and in preventing the emergence of antimicrobial resistance [18,19,20].

Currently, drinking water has been recognized as an important area of intervention [8], and according to the EU regulation 183/2005, water treated with decontaminants may be used [21]. There are many chemical solutions on the market, which are used when animals are absent and are designed for aggressive cleaning of the drinking water lines, while others are administered to drinking water when animals are present in order to maintain quality and improve the health and performance of the flock [22,23,24]. Among others, commercial products based on organic and inorganic acids are gaining popularity since their components are not considered particularly harmful to the ecosystem and promote antibacterial activity [20,25]. Inorganic acids (such as H_2_O_2_) and organic acids (like formic, acetic, propionic, caproic, caprylic, and butyric acids and their salts) could act as efficient tools in hindering the spread of pH-sensitive zoonotic bacteria like *Campylobacter* spp. and *Salmonella* spp. among the flock [8,26]. In addition, acidic blends seem to positively affect the performance, health, and welfare of poultry by improving nutrient digestibility, managing gastric secretions accompanied by histological alterations, and stimulating the gut immune system of birds [20,25].

There are many knowledge gaps concerning the ability of bacteria to develop resistance to these alternative antimicrobials [27,28,29,30]. There is evidence that residual levels of applied acidic blends or disinfectants may trigger adaptation to oxidative stress by genetic mutations and/or horizontal transfer of antimicrobial resistance genes among the exposed bacteria [29,30]. However, the lack of an established guideline to test their efficacy, as well as the absence of MIC breakpoints, makes it challenging to classify bacteria as resistant or susceptible to these alternative biocides and highlights the significance of future research [29,31]. In addition, understanding how these products act against common poultry pathogens in vitro could be helpful in designing a holistic and economically viable dosage scheme for further in vivo and in-field investigations. To date, there are only a few available studies on the antimicrobial activity of commercial products [32,33,34,35], and the results are non-comparative due to variable examination methods, tested strains, and culture conditions [36].

The aim of the present study was to determine, in vitro, the minimal inhibitory concentration (MIC) of nine commercial water disinfectants, acidifiers, and glyceride blends against a panel of the most important poultry zoonotic bacteria. The selected products (Cid 2000™, Aqua-clean^®^, Virkon^®^ S, Agrocid Super™Oligo, Premium acid, Ultimate acid, CFC Floramix, FRA^®^ LAC34, and FRA^®^ Gut Balance) are commonly used in poultry farms, whereas pathogens like *Campylobacter* spp., *Salmonella* spp., *Escherichia* spp., *Staphylococcus* spp., and *Listeria* spp. are frequently isolated from poultry and their products in the market.

## 2. Materials and Methods

### 2.1. Tested Products

A total of nine commercial products that have EU approval for application in the drinking water of poultry were investigated for their potential inhibitory effect against the most important zoonotic bacteria of poultry. According to the technical data and their chemical formulas, tested products were grouped into three categories *:

(1) **Water disinfectants**: Cid 2000™ (CID LINES N.V., Leper, Belgium), Aqua-clean^®^ (Kanters, Lieshout, The Netherlands), and Virkon^®^ S (Lanxess, Belgium).
(2) **Water acidifiers**: Agrocid Super™Oligo (Cid lines N.V., Leper, Belgium), Premium acid, Ultimate acid (Kanters, Lieshout, The Netherlands).
(3) **Water glyceride blends**: CFC Floramix (Kanters, Lieshout, The Netherlands), FRA^®^ LAC34, and FRA^®^ Gut Balance (Framelco, Raamsdonksveer, The Netherlands).

All products were water-soluble and liquid at ambient temperature, except for Virkon^®^ S, which was provided in a pink-colored powder form. The active ingredients for each product and the maximum dosage recommended by the manufacturer are shown in Table 1.

* Many of the commercial formulas contain ingredients that may belong to more than one product category, so the distinction used in this study cannot be absolute.

### 2.2. Tested Bacterial Strains

A total of 13 bacterial strains, seven Gram-negative (G-) and six Gram-positive (G+), were selected for this study. All tested bacteria are of great importance to the poultry industry and public health, with some exhibiting antibiotic resistance. *Campylobacter jejuni* S1 (S1: Strain 1) and *Campylobacter coli* S1 are field isolates of avian and pork origin, respectively, provided by the *Campylobacter* strain collection of the Greek National Reference Laboratory for *Campylobacter*, Ioannina, Greece. These isolates were collected between 2018 and 2019 on the basis of epidemiological surveillance. *Campylobacter jejuni* S2 (S2: Strain 2) was isolated in 2019 from a commercial poultry slaughterhouse as part of the routine monitoring program and was identified by PCR. *Escherichia coli* ATCC 25922 is a reference strain of clinical origin, that is proposed for antimicrobial susceptibility testing, by the Clinical and Laboratory Standards Institute (CLSI) and the European Committee on Antimicrobial Susceptibility Testing (EUCAST). *Escherichia coli* ATCC 11303 is a culture strain used as a bacteriophage host and as a model organism for *Escherichia coli* protocols. *Salmonella* Typhimurium DT 120 is a pathogenic strain isolated during a salmonellosis outbreak in Denmark and has been linked to the consumption of turkey meat. *Salmonella* Typhimurium U292 is a pathogenic strain isolated during a salmonellosis outbreak in Denmark from a patient suffering from gastroenteritis. *Staphylococcus aureus* DSM 25629 is a pathogenic strain of unknown origin exhibiting multidrug resistance. Staphylococcus aureus DSM 102262 is a multidrug-resistant pathogenic strain isolated from a human patient in North Korea, whereas *Staphylococcus aureus* S1 is a wild strain isolated from a surface in a commercial poultry slaughterhouse. *Listeria monocytogenes* Scott A is a serovar 4b clinical isolate from the 1983 listeriosis outbreak in Massachusetts, USA, and is widely used as a reference pathogenic strain. *Listeria innocua* ATCC 33090 is a reference strain isolated from a cow brain that is used as a quality control strain in several microbiological tests involving *Listeria*. *Listeria monocytogenes* S1 is a serotype 1/2a wild strain isolated from raw chicken meat. All the tested strains are represented in Table 2.

### 2.3. Preparation of the Tested Inoculums

All tested strains were maintained at −80 °C in a 13% (*w/w*) glycerol broth until use. Fresh cultures were prepared in Brain Heart Infusion broth (BHI, CM1135, Oxoid Ltd., Basingstoke, UK) and incubated under suitable conditions (*E. coli*, *S*. Typhimurium, *S. aureus*, and *Listeria* spp. were incubated at 37 °C for 24 h; *C. jejuni* and *C. coli* were incubated at 42 °C for 48 h under microaerobic conditions). Before use, each strain was inoculated on a Petri dish containing BHA or cation-adjusted MH agar (B11438, Becton Dickinson, Franklin Lakes, NJ, USA). For the *Campylobacter* strains, MH agar was supplemented with 5% horse blood (Oxoid, CM 129). After incubation, cultures were examined for typical morphological, microscopical, and biochemical characteristics for each pathogen, and a second culture was prepared (working culture). Colonies from the working culture were used to prepare a 0.5 McFarland suspension in MH broth and were further diluted to obtain a working solution of 10^6^ CFU of bacteria/mL.

### 2.4. MIC Assay

**Figure 1:**
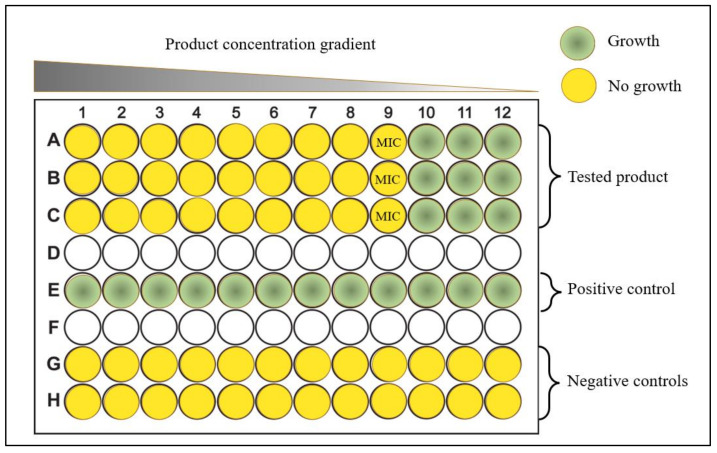
Illustrative scheme of the micro-dilution MIC method that was used for the investigation of the antibacterial activity of commercial water disinfectants, acidifiers, and glyceride blends against the most important poultry zoonotic bacteria.

The determination of the Minimal Inhibitory Concentration (MIC) of the products was performed according to CLSI standards M07-A10 [37] and M100-S28 [38], with modifications. Each product was tested by the microdilution MIC method (micro-MIC) in concentrations ranging from 0.002 to 1.136% *v/v* (or *w/v*). For each microorganism, a flat-bottomed 96-well polystyrene microtiter plate (TC-Platte 96-Well, Standard, F) was used and filled with cation-adjusted Mueller–Hinton broth (CM0405, Oxoid Ltd., Basingstoke, UK). The product was added, in triplicate, in two-fold serial dilutions within the microtiter plates, followed by the inoculation of the wells with the bacterial strain. Negative and positive control wells were prepared with only the tested product or the culture medium, respectively (Figure 1). Each microplate was sealed and incubated at 37 °C for 24 h. For *Campylobacter* spp., incubation was performed at 42 °C under a microaerophilic atmosphere (approximately 5% O_2_, 10% CO_2_, and 85% N_2_) for 48 h. The growth was evaluated, after 24 h (48 h for *Campylobacter* spp.), by visual inspection of turbidity as an indicator of microbial growth. MIC values were determined as the lowest antimicrobial agent concentration (%*v/v* or *w/v*) that inhibited microbial growth.

### 2.5. Statistical Analysis

Raw data derived from MIC measurements were processed using methods of biostatistics with the SPSS^®^ Statistics Ver. 25 (IBM Corp, Armonk, NY, USA). For each product, the MIC score against a tested pathogen was interpreted as the mean MIC score (%*v/v* ± standard deviation) of the individual score of each triplicate. In order to obtain a more conclusive approach, for bacterial species for which we tested more than one isolate, the data were grouped and statistical analysis was performed with GraphPad Prism (version 9.5.0 for Windows^®^, GraphPad Software, San Diego, CA, USA). Specifically, multiple comparisons between treatment means were made with non-parametric Mann–Whitney tests. The significance level was set at *p* ≤ 0.05.

## 3. Results

**Figure 2:**
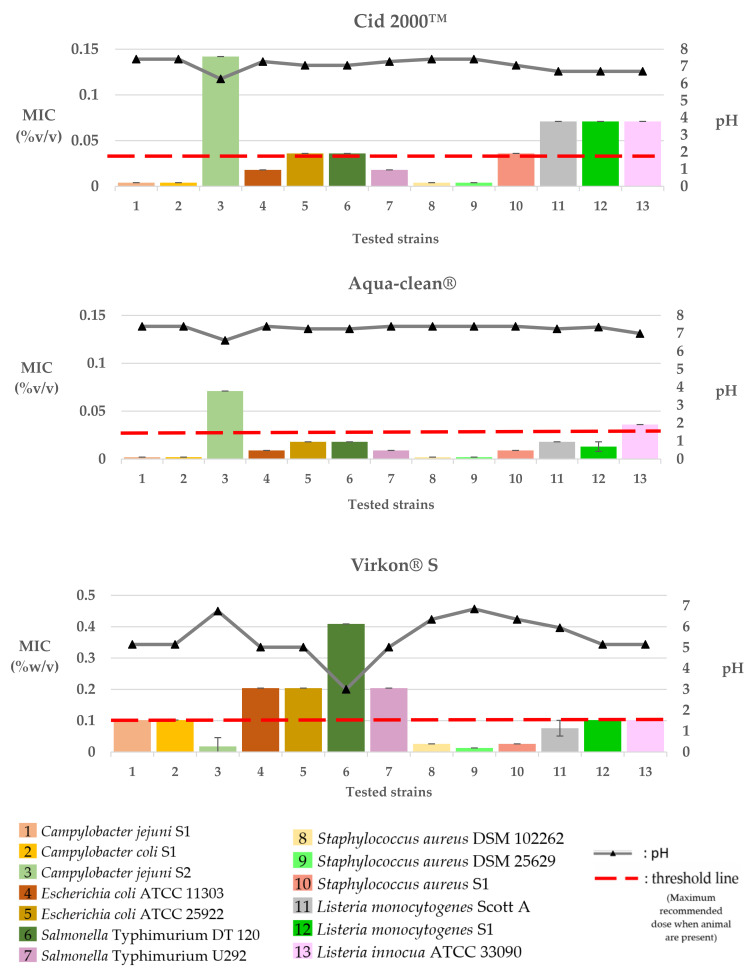
Graphical portrayal of the recorded MICs (columns; mean %*v/v* ± SD) of the tested commercial water disinfectants on the tested bacteria strains and of the pH value (triangles on the pH line) of the broth medium in each MIC.

**Figure 3:**
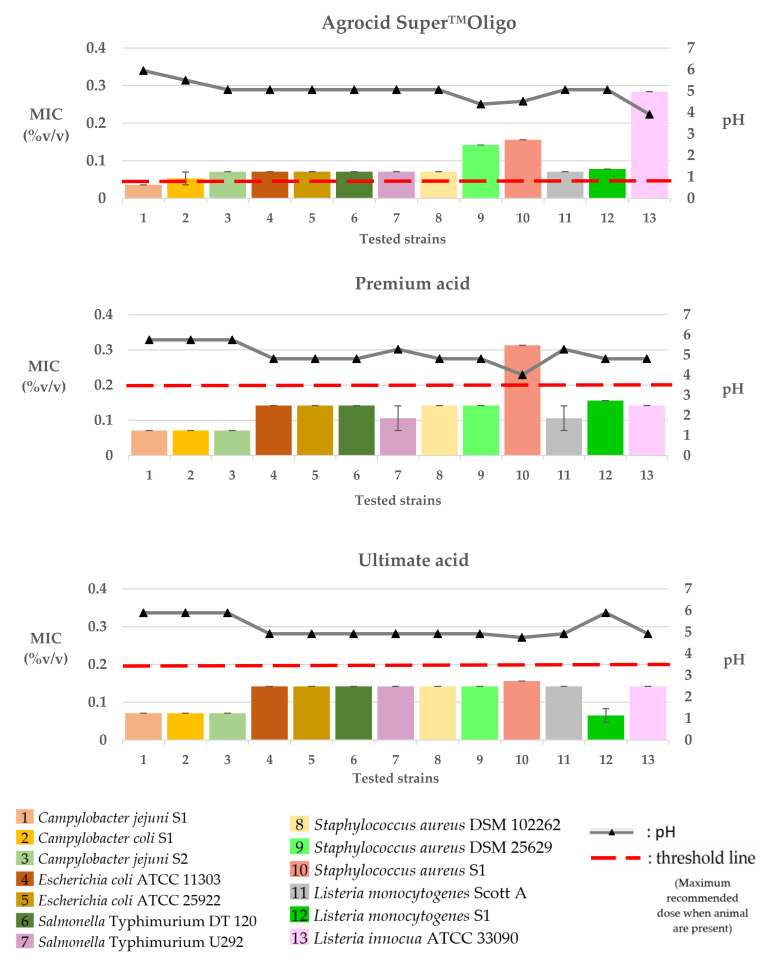
Graphical portrayal of the recorded MICs (columns; mean %*v/v* ± SD) of the tested commercial water acidifiers on the tested bacteria strains and of the pH value (triangles on the pH line) of the broth medium in each MIC.

**Figure 4:**
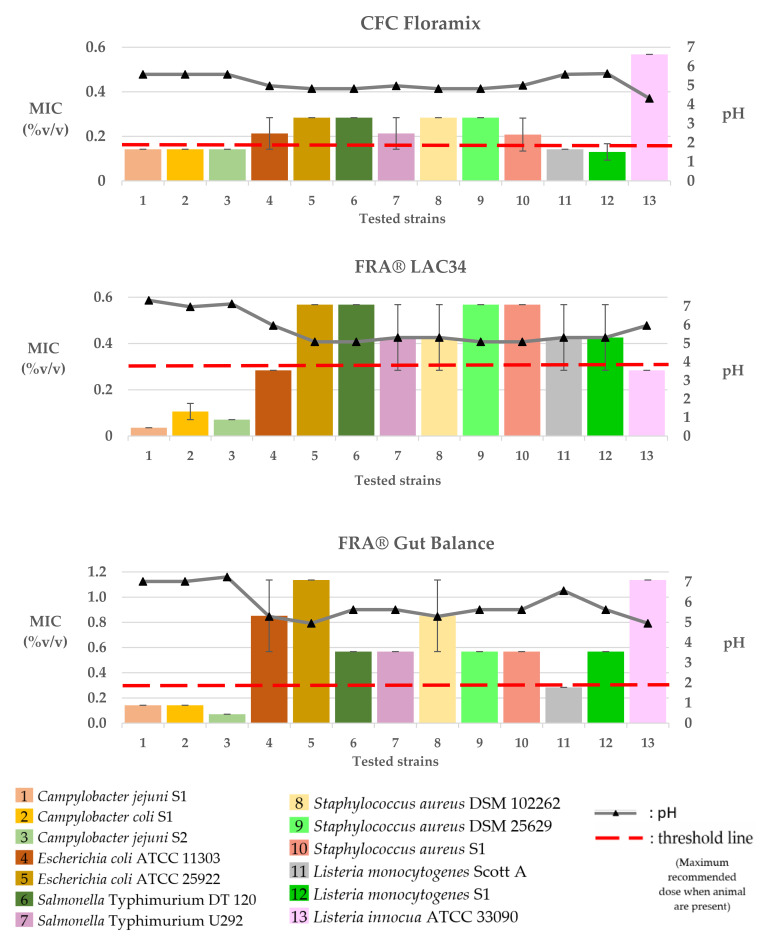
Graphical portrayal of the recorded MICs (columns; mean %*v/v* ± SD) of the tested commercial water glycerides blends on the tested bacteria strains and of the pH value (triangles on the pH line) of the broth medium in each MIC.

**Figure 5:**
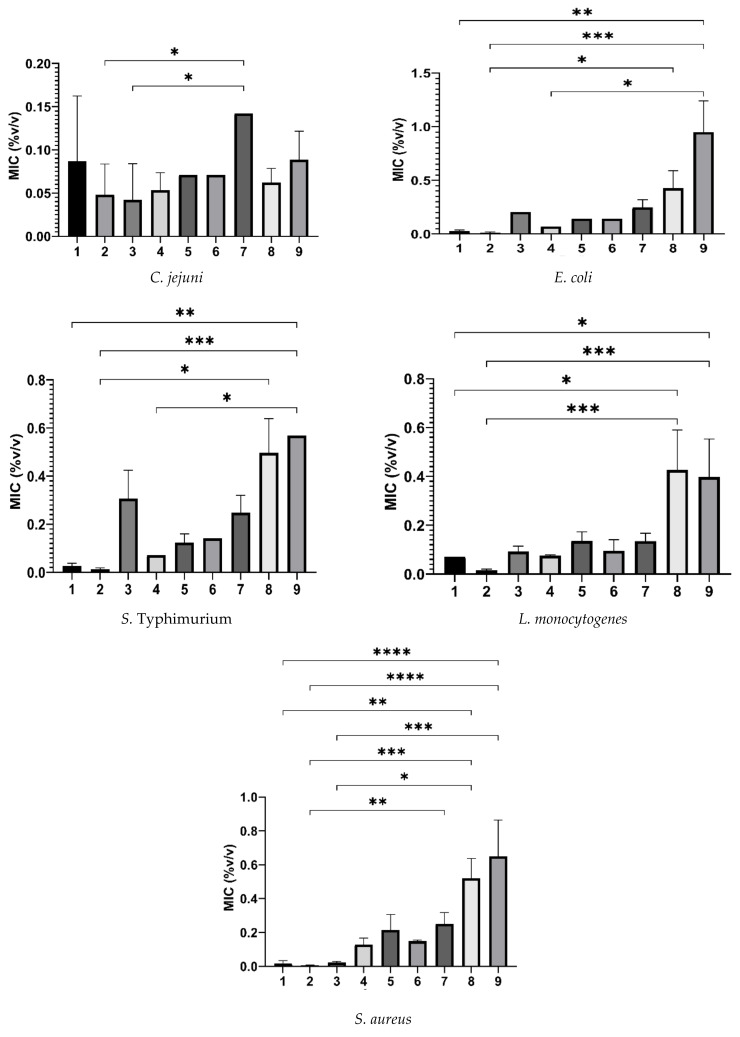
A panel of graphical presentations received from statistical analysis of the MICs (columns; mean %*v/v* ± SD) of the tested commercial products (1–9) against the tested bacteria (for bacterial species for which we tested more than one isolate) by the GraphPad Prism (version 9.5.0 for Windows^®^, GraphPad Software, San Diego, CA, USA). **1**: Cid 2000™; **2**: Aqua-clean^®^; **3**: Virkon^®^ S; **4**: Agrocid Super™Oligo; **5**: Premium acid; **6**: Ultimate acid; **7**: CFC Floramix; **8**: FRA^®^ LAC34; **9**: FRA^®^ Gut Balance. *: *p* < 0.05, **: *p* < 0.01, ***: *p* < 0.001, ****: *p* < 0.0001.

The MIC values of the tested commercial poultry products against the selected bacteria strains are presented in Table 3, as well as in Figure 2, Figure 3, Figure 4 and Figure 5.

### 3.1. Water Disinfectants

The MIC values of Cid 2000™ against the tested bacteria were 0.004% *v/v* for *S. aureus* (DSM 25629 and DSM 102262) and *Campylobacter* (*C. jejuni* S1 and *C. coli* S1) and 0.018 or 0.036% *v/v* for *E. coli* (ATCC 11303 and ATCC 25922), *S.* Typhimurium (U292 and DT 120), and *S. aureus* S1. The higher MIC values of Cid 2000™ were 0.071% *v/v* for *Listeria* strains (*L. monocytogenes* Scott A and S1, *L. innocua*; ATCC 33090) and 0.142% *v/v* for *C. jejuni* S2. Following the same pattern, the lowest MIC values (0.002% *v/v*) of Aqua-clean^®^ were recorded in *S. aureus* (DSM 25629 and DSM 102262) and *Campylobacter* (*C. jejuni* S1 and *C. coli* S1), followed by MICs of 0.009 or 0.018% *v/v* for *E. coli* (ATCC 11303 and ATCC 25922), *S.* Typhimurium (U292 and DT 120), *S. aureus* S1, and *L. monocytogenes* (Scott A and S1). The higher MIC values of Aqua-clean^®^ were 0.0036% *v/v* for *L. innocua* ATCC 33090 and 0.071% *v/v* for *C. jejuni* S2. Finally, Virkon^®^ S recorded MIC values ranging from 0.013–0.409% *w/v* among the tested pathogens. The lowest MIC values of 0.013 or 0.026% *w/v* were recorded against *S. aureus* (DSM 25629, DSM 102262, and S1), followed by 0.018% *w/v* for *C. jejuni* S2 and 0.076 or 0.102% *v/v* against *Listeria* strains (*L. monocytogenes*; Scott A and S1, *L. innocua*; ATCC 33090) and *Campylobacter* strains (*C. jejuni* S1 and *C. coli* S1). Higher concentrations of the product (0.204 *w/v*) were required to inhibit the growth of *E. coli* (ATCC 11303 and ATCC 25922) and *S.* Typhimurium U292, whereas even higher concentrations (0.409%) were required for the inhibition of *S.* Typhimurium DT 120.

### 3.2. Water Acidifiers

The MIC values of Agrocid Super™Oligo were 0.036 or 0.071% *v/v* for *Campylobacters* (*C. jejuni* S1 and S2, *C. coli* S1) and 0.071% *v/v* for *E. coli* (ATCC 11303 and ATCC 25922), *S.* Typhimurium (U292 and DT 120), *Listeria* strains (*L. monocytogenes* Scott A and S1), and *S. aureus* DSM 102262. Higher MICs of 0.142% *v/v* were recorded on *S. aureus* (DSM 25629 and S1), whereas the MIC of the product for *L. innocua* ATCC 33090 was 0.284% *v/v*. Premium acid recorded MICs ranging from 0.071 to 0.568% *v/v*. The lower MIC values (0.071% *v/v*) were recorded against *Campylobacters* (*C. jejuni* S1 and S2, *C. coli* S1), followed by MICs of 0.142% *v/v* for *S.* Typhimurium (U292 and DT 120), *E. coli* (ATCC 11303 and ATCC 25922), *S. aureus* (DSM 25629 and DSM 102262), and *Listeria* strains (*L. monocytogenes* S1, *L. innocua*; ATCC 33090). Higher concentrations of 0.284–0.568% *v/v* were required for the inhibition of *S. aureus* S1. Finally, the MIC values of the Ultimate acid were 0.036 or 0.071% *v/v* for *L. monocytogenes* S1 and *Campylobacter* (*C. jejuni* S1 and S2, *C. coli* S1) and 0.142% *v/v* for the rest of the tested pathogens.

### 3.3. Water Glyceride Blends

The MIC values of CFC Floramix were approximately 0.142% *v/v* for *Listeria monocytogenes* (Scott A and S1) and *Campylobacter* (*C. jejuni* S1 and S2, *C. coli* S1) and 0.284% *v/v* for *E. coli* (ATCC 11303 and ATCC 25922), *S.* Typhimurium (U292 and DT 120), and *S. aureus* (DSM 25629, DSM 102262, and S1). Finally, higher concentrations of the product (0.568% *v/v*) were required for the inhibition of *L. innocua* ATCC 33090. FRA^®^ LAC34 recorded MICs ranging from 0.036 to 0.568% *v/v* among the tested isolates. The lower MIC values of 0.036% *v/v*, 0.071% *v/v*, or 0.142% *v/v* were recorded against *C. jejuni* S1, S2, and *C. coli*, respectively. Higher concentrations of the product (0.284% *v/v*) were required to inhibit the growth of *E. coli* ATCC 11303 and *L. innocua* ATCC 33090, followed by MICs of 0.284–0.568% *v/v* for *S.* Typhimurium U292, *S. aureus* DSM 102262, and *Listeria* strains (*L. monocytogenes* Scott A and S1) and 0.568% *v/v* for *E. coli* ATCC 25922, *S.* Typhimurium DT 120, and *S. aureus* (DSM 25629 and S1). The MIC values of FRA^®^ Gut Balance were 0.071% v/v for *C. jejuni* S2, 0.142% *v/v* for *C. jejuni* S1 and *C. coli* S1, 0.284% *v/v* for *L. monocytogenes* Scott A, 0.568% *v/v* for *S. aureus* (DSM 25629 and S1), *L. monocytogenes* S1, and *S.* Typhimurium (U292 and DT 120) and 0.568–1.136% *v/v* for *E. coli* ATCC 11303 and *S. aureus* DSM 102262. Finally, higher MICs of 1.136% *v/v* were recorded against *E. coli* ATCC 25922 and *L. innocua* ATCC 33090.

The pH values of the culture broth at various concentrations of the tested products are shown in Figure 2, Figure 3 and Figure 4.

**Table 3:** The minimum inhibitory concentrations (*v/v* *) of the tested commercial products on the tested bacteria strains ($\bar{x}$ ± SD).

	Water Disinfectants			Water Acidifiers			Water Glyceride Blends		
Tested Strain	Cid 2000™	Aqua-Clean^®^	Virkon^®^ S	Agrocid Super™Oligo	Premium Acid	Ultimate Acid	CFC Floramix	FRA^®^ LAC34	FRA^®^ Gut Balance
*C. jejuni* S1	0.004 ± 0.000	0.002 ± 0.000	0.102 ± 0.000	0.036 ± 0.000	0.071 ± 0.000	0.071 ± 0.000	0.142 ± 0.000	0.036 ± 0.000	0.142 ± 0.000
*C. coli* S1	0.004 ± 0.000	0.002 ± 0.000	0.102 ± 0.000	0.053 ± 0.017	0.071 ± 0.000	0.071 ± 0.000	0.142 ± 0.000	0.106 ± 0.035	0.142 ± 0.000
*C. jejuni* S2	0.142 ± 0.000	0.071 ± 0.000	0.018 ± 0.028	0.071 ± 0.000	0.071 ± 0.000	0.071 ± 0.000	0.142 ± 0.000	0.071 ± 0.000	0.071 ± 0.000
*E. coli* ATCC 11303	0.018 ± 0.000	0.009 ± 0.000	0.204 ± 0.000	0.071 ± 0.000	0.142 ± 0.000	0.142 ± 0.000	0.213 ± 0.071	0.284 ± 0.000	0.852 ± 0.284
*E. coli* ATCC 25922	0.036 ± 0.000	0.018 ± 0.000	0.204 ± 0.000	0.071 ± 0.000	0.142 ± 0.000	0.142 ± 0.000	0.284 ± 0.000	0.568 ± 0.000	1.136 ± 0.000
*S*. Typhimurium DT120	0.036 ± 0.000	0.018 ± 0.000	0.409 ± 0.000	0.071 ± 0.000	0.142 ± 0.000	0.142 ± 0.000	0.284 ± 0.000	0.568 ± 0.000	0.568 ± 0.000
*S*. Typhimurium U292	0.018 ± 0.000	0.009 ± 0.000	0.204 ± 0.000	0.071 ± 0.000	0.106 ± 0.035	0.142 ± 0.000	0.213 ± 0.071	0.426 ± 0.142	0.568 ± 0.000
*S. aureus* DSM 102262	0.004 ± 0.000	0.002 ± 0.000	0.026 ± 0.000	0.071 ± 0.000	0.142 ± 0.000	0.142 ± 0.000	0.284 ± 0.000	0.426 ± 0.142	0.852 ± 0.284
*S. aureus* DSM 25629	0.004 ± 0.000	0.002 ± 0.000	0.013 ± 0.000	0.142 ± 0.000	0.142 ± 0.000	0.142 ± 0.000	0.284 ± 0.000	0.568 ± 0.000	0.568 ± 0.000
*S. aureus* S1	0.036 ± 0.000	0.009 ± 0.000	0.026 ± 0.000	0.156 ± 0.000	0.313 ± 0.000	0.156 ± 0.000	0.208 ± 0.074	0.568 ± 0.000	0.568 ± 0.000
*L.monocytogenes* Scott A	0.071 ± 0.000	0.018 ± 0.000	0.076 ± 0.025	0.071 ± 0.000	0.106 ± 0.035	0.142 ± 0.000	0.142 ± 0.000	0.426 ± 0.142	0.284 ± 0.000
*L.monocytogenes* S1	0.071 ± 0.000	0.013 ± 0.005	0.102 ± 0.000	0.078 ± 0.000	0.156 ± 0.000	0.065 ± 0.018	0.130 ± 0.037	0.426 ± 0.142	0.568 ± 0.000
*L. innocua* ATCC 33090	0.071 ± 0.000	0.036 ± 0.000	0.102 ± 0.000	0.284 ± 0.000	0.142 ± 0.000	0.142 ± 0.000	0.568 ± 0.000	0.284 ± 0.000	1.136 ± 0.000

MIC values are represented as % v/v * concentration of the product in the Mueller–Hinton broth. * Virkon S was the only product which was in powder water-soluble form, and MIC values are therefore represented as % *w/v*.

## 4. Discussion

Water sanitation and/or acidification is a promising practice for lowering the use of antibiotics in human and veterinary medicine [18,19,20]. However, the existence of knowledge gaps on the antibacterial activity of the commercially available water disinfectants, acidifiers, and glyceride blends, as well as the potential for microbes to develop resistance to these alternative biocides [27,28,29,30], emphasizes the significance of future research [39].

In the present study, we performed an in vitro screening of the ability of nine commercial water disinfectants, acidifiers, and glyceride blends to inhibit seven bacterial pathogens of importance to both human and poultry health. Most of the tested products effectively inhibited the growth of the selected microorganisms at the manufacturer’s recommended dosages (Figure 2, Figure 3 and Figure 4). Products of the “water disinfectants” group (Cid 2000™ and Aqua-clean^®^) displayed the highest antimicrobial capacity (Figure 2 and Figure 5), followed by those of the “water acidifiers” group (Agrocid Super™Oligo, Premium acid, and Ultimate acid) (Figure 3 and Figure 5), Virkon^®^ S, and finally those of the “Water glycerides blends” group (Figure 4 and Figure 5) (CFC Floramix, FRA^®^ LAC34, and FRA^®^ Gut Balance). These results might be reflected in the susceptibility of each microorganism and/or the unique synthesis of each commercial product, as well as its capacity to modify the physicochemical character of the broth medium [40].

Cid 2000™ and Aqua-clean^®^ exhibited extremely low MICs (0.002–0.142% *v/v*) against the tested pathogens. In both products, the main active ingredient is hydrogen peroxide (H_2_O_2_). Hydrogen peroxide is a weak acid that readily passes through cell membranes and can denature enzymes and proteins or inhibit other biological activities in a wide range of micro-organisms, including bacteria, viruses, and fungi [41]. Since hydrogen peroxide quickly breaks down into water and oxygen, most commercial formulae, such as Cid 2000™ and Aqua-clean^®^, use additional ingredients (organic acids such as peracetic and ascorbic acids; heavy metal ions such as those of silver or copper) for stabilization and/or for enhancing effectiveness [42].

Commercial blends of hydrogen peroxide, stabilized by peracetic and/or acetic acid (such as Cid 2000™), are considered potent oxidizing agents and are widely employed by the poultry industry since their components are not considered harmful to the ecosystem [43]. In the present study, Cid 2000™ was effective by inhibiting the growth of the tested micro-organisms recording MICs of 0.004–0.142% *v/v*. In agreement with our findings, Hancock et al. (2007) reported that Cid 2000™ is highly effective in reducing the total anaerobic and aerobic bacterial counts as well as the level of yeasts and molds when applied in 2% *v*/*v* water solution [44]. Additionally, hydrogen peroxide and peracetic acid blends were effective when applied in poultry chillers since they reduced the number of positive *Salmonella* spp. and *Campylobacter* spp. carcasses by 92% and 43%, respectively [45]. Briñez et al. (2006) investigated the in vitro antibacterial capacity of peracetic acid and hydrogen peroxide, according to the EU standard suspension test UNE-EN-1276, against pathogenic and non-pathogenic strains of *Listeria* spp., *E. coli*, and *Staphylococcus* spp. Researchers noted that the tested *Staphylococcus* spp. strains were more resistant than the strains of *E. coli* and *Listeria* spp. at low concentrations of this disinfectant [46]. The results of this study do not support this hypothesis since Cid 2000™ was highly effective against the tested ATCC strains of *Staphylococcus* spp. with an MIC of 0.004% *v/v*, which was lower than those described for the tested *E. coli* (0.018–0.036% *v/v*) and *Listeria* strains (0.071% *v/v*). However, the 10-fold higher concentration (0.036% *v/v*) required for Cid 2000™ to inhibit the growth of *S. aureus* S1 (Figure 2), compared to the reference ATCC *S. aureus* strains (0.004% *v/v*), could: be evidence of resistance to this disinfectant group; due to the strain–species variability; or support the hypothesis that wild isolates like *S. aureus* S1. are more resistant to biocides compared to laboratory strains.

In our study, Aqua-clean^®^, a blend of hydrogen peroxide stabilized by a silver complex, exhibited the lowest MIC values against most of the tested micro-organisms (Figure 2 and Figure 5). It was previously suggested that adding silver enhances the biocidal activity of hydrogen peroxide [47,48]. It is hypothesized that although hydrogen peroxide mainly affects the lipids, proteins, and nucleic acids of bacteria, silver primarily affects sulfhydryl protein groups or/and provides a synergistic effect by causing electrostatic interactions at the bacterial cell surface [48]. In concordance with our results, El-Gohary et al. (2020) reported the high antibacterial capacity of a novel hydrogen peroxide product, combined with silver nanoparticles, against a wide range of isolates, including strains of *S*. Typhimurium, *E. coli* O157:H7, and *L. monocytogenes* [49]. However, despite the promising antibacterial capacity of Aqua-clean^®^, the inclusion of silver particles in the diet of animals should be carefully considered since there is a trend for the elimination of heavy metals use [50].

Health organizations actively support the use of hydrogen peroxide in animal drinking water as an efficient defense against zoonotic pathogens like *C. jejuni* [8]. The results of our investigation might provide credence to this recommendation since most of the tested *Campylobacter* strains were highly susceptible (MICs: 0.002–0.004% *v/v*) in both commercial blends of H_2_O_2_. Interestingly, both products required higher concentrations (Cid 2000™: 0.142% *v/v*; Aqua-clean^®^: 0.071% *v/v*) to inhibit the growth of *C. jejuni* S2 (Figure 2), in contrast to those required for the other *Campylobacter* strains (Cid 2000™: 0.004% *v/v*; Aqua-clean^®^: 0.002% *v/v*). *C. jejuni* S2 has been isolated from a commercial poultry slaughterhouse that uses hydrogen peroxide products in its sanitation protocol. It is reported that residual levels of applied disinfectants and/or their by-products may be detected in the food processing environment and the drinking line systems and may trigger adaptation to oxidative stress by genetic mutations and/or horizontal transfer of antimicrobial resistance genes among the exposed bacteria [30]. This scenario could support the 20-fold larger concentrations required for hydrogen peroxide-based products to inhibit *C. jejuni* S2 compared to concentrations required for the other *C. jejuni* strain (S1) inhibition. However, it is challenging to classify bacteria as resistant or susceptible to these alternative biocides due to the lack of an established guideline to test their efficacy and the absence of MIC breakpoints [29,36].

Virkon^®^ S, a commercial disinfectant blend of peroxides, inhibited the growth of the tested pathogens recording MICs ranging from 0.013 to 0.409% *w/v*. It is suggested that its efficacy might be due to its high potassium concentration and/or combination with other active chemical agents, such as sulfamic acid and sodium dodecylbenzene sulfonate [51]. Our results demonstrated that Virkon^®^ S recorded higher MIC mean values against the tested Gram-negative isolates (Figure 2), indicating that it was more efficient at suppressing the growth of the Gram-positive bacteria. This finding is consistent with that of earlier researchers and may be supported by the fact that the outer layer of Gram-negative bacteria is composed primarily of lipopolysaccharides, which may inhibit the effectiveness of many biocides that target the bacterial cell membrane [33,52], such as Virkon^®^ S in this instance.

Like most commercially available formulas, water acidifiers used in this study are blends of organic acids (mainly SCFA and/or MCFA), whereas additional ingredients like zinc or copper chelates, zinc chloride, or oligofructose sirup are included in some formulas to improve effectiveness. SCFA (C1–C6), like formic, propionic, acetic, or butyric acids, are the components that are highly concentrated in the products Agrocid Super™Oligo, Premium acid, and Ultimate acid, and to that end, could be responsible for their antimicrobial activity in vitro. For SCFA, various mechanisms for their antibacterial mode of action have been suggested, with their main mode of action being the ability of the undissociated forms to penetrate through the semipermeable membrane of the bacteria cell and acidify cell cytoplasm, resulting in the inhibition of bacterial growth [19,26]. In the present study, all the water acidifiers exhibited antibacterial activity against all the tested pathogens in concentrations of 0.071–0.458% *v/v*. Moreover, a correlation between the antibacterial activity of water acidifiers with their capacity to regulate the pH of the culture medium close to 5 was noted (Figure 3 and Figure 4). Perhaps the neutralization of the inhibitory effect of water sanitizers is accompanied by an increase in pH values as well. In agreement with previous investigators [53], we concluded that pH is a primary determinant of effectiveness because it affects the concentration of the undissociated or dissociated form of the acid and, therefore, its antibacterial capacity [53,54].

Organic acid-based commercial products for water application are frequently referred to as water acidifiers. While it is reported that adding organic acids to drinking water reduces its pH, this is typically dependent on the chemical characteristics of the drinking water of each farm [55]. Organic matter, chlorination, and drinking water contamination can influence commercial formulations’ effectiveness [56]. Thus, recommended dosages would appear to be somewhat below the level required to appreciably “acidify”, and in many cases, the term water “acidifier” may be misleading. In addition, the antibacterial activity of water acidifiers might be limited to drinking water or the birds’ crop as the buffering effect of the gastric environment reduces its bioaccumulation in the intestines [57].

In comparison to products made with free SCFAs, next-generation products that incorporate SCFA with their glycerol ester forms have noteworthy advantages [58]. Glycerides of SCFA are more resistant to chemical changes than free SCFAs and have a milder odor, making them desirable alternatives to antibiotics in animal feed or drinking water [59]. Additionally, organic acid ester forms are broken down and absorbed as lipids, ensuring that they are protected by the acidic pH in the stomach and successfully release their antimicrobial properties into the intestine [58,59].

As a general rule, the antibacterial activity of a fatty acid is increased when it is esterified [59]. In the current study, the products that belonged to the “glycerides group” were composed of various concentrations of mono- and diglycerides of fatty acids (propionic, butyric, and/or caprylic acids), in combination with free organic acids in some products. Our results demonstrated that all the glyceride blend products exhibited variable inhibitory effects on the tested bacteria, recording MICs ranging from 0.036 to 0.587% *v/v*. The *Campylobacter* strains used were highly susceptible to the “glyceride blends” compared to the other tested bacteria (Figure 4). It is believed that the O-side chains of lipopolysaccharides in the outer membrane of Gram-negative bacteria comprise an effective barrier for hydrophilic molecules, such as monoglycerides [35]. However, the outer membrane of *Campylobacter* spp. expresses lipooligosaccharides that lack the O-side chain [60] and thus could be more susceptible to glyceride blends than *E. coli* and *S*. Typhimurium.

Several research studies have focused on determining the antimicrobial effectiveness of various pure ingredients subjected to commercial formulas. However, only a few studies [32,33,34,35] are available on the antimicrobial capacity of commercially available formulas, which are commonly blends of ingredients. In addition, many of the existing studies do not provide information about the composition of the products; therefore, the direct comparison of literature with the data in our study was difficult. The lack of an appropriate testing protocol that closely recreates the industry settings could explain why similar (but not identical) chemical formulations have very different efficacy against bacteria when evaluated [31].

In this study, the MIC values of some of the products against the tested pathogens were considerably lower, assuming that the recommended concentrations for use in the field are high enough (Figure 2, Figure 3 and Figure 4). However, we should keep in mind that MIC value is only indicative since it represents only the direct effect of the tested product against the tested pathogen. It does not consider the indirect effect that could be had on the intestinal microbiota, feed ingredients, immune system, and intestinal digesta [25,57,59]. In addition, the biocide efficiency could be affected by various parameters, including concentration, pH, formulation, temperature, presence of organic matter and biofilms in the drinking lines, as well as contact time [54,55,61]. Finally, the relatively few isolates that were investigated limit the interpretability of our results to single bacterial species.

For some products, such as water acidifiers and glyceride blends, chemical formulations and manufacturer recommendations are promising for additional indirect antimicrobial effects under in vivo applications [25,57,59], whereas for other products, such as hydrogen peroxide-based formulas, no prior data are available. It is reported that applying organic acid blends through drinking water could reduce the pH in the bird’s crop and thus serve as an effective barrier in the growing and spreading of pH-sensitive bacteria like *Salmonella* and *Campylobacter* [19,26]. Additionally, organic acids and their glyceride forms are well documented for their effectiveness in enhancing gut histomorphology, boosting gut immunity, and altering the physicochemical characteristics of intestinal content, which collectively result in changes in the composition of the gut microbiome [25,57,59,62].

In this study, the classification of the tested products was based on their active compounds and the recommendations of the manufacturer. However, many of the commercial formulas contain ingredients that may belong to more than one product category, so the distinction used in this study cannot be absolute. Finally, it is obvious that not all water additives will produce the required or desired results under in-field application, as their action is highly dependent on factors like the chemical composition of the water on the farm, the feed composition, the dosage scheme, the age of the birds, and the overall management of the flock [63].

## 5. Conclusions

The method utilized in this study can determine the MICs of nine commercial poultry products for water application to a panel of microorganisms in a single test and will be useful in evaluating the efficacy of biocides used in the field. Most of the tested products showed promising antibacterial activity, and as a result, they would be good candidates for lowering in vivo pathogen colonization and reducing the emergence of antimicrobial resistance in poultry. According to their synthesis, it is expected for some of the products to exhibit high potential in the upper gastrointestinal tract or the stomach, whereas others, such as glyceride blends, are expected to deliver benefits in the small intestine of animals. However, it is crucial to highlight that due to the lack of interfering substances in the in vitro testing, conclusions may under- or overestimate the effectiveness of the products. Further in vivo and in-field studies are required to provide relevant information for establishing the optimal concentration for pathogen control for each product. Finally, a holistic approach is required to determine the actual impact of these products on the health and welfare of birds and on the productivity of commercial poultry operations.

## Figures and Tables

**Table 1:** Information on the synthesis and the recommended dose of the selected commercial products.

PC	Tested Product	Form	Active Ingredients (% Concentration)	r. Dose ^1^
**Water Disinfectants**	Cid 2000™	Liquid	Hydrogen peroxide (15–30%), peracetic acid (5–15%), acetic acid (5–15%)	0.040%
	Aqua-clean^®^	Liquid	Hydrogen peroxide (25–50%), complexed silver	0.025%
	Virkon^®^ S	Solid	Pentapotassium bis (peroxymonosulphate) bis (sulphate) (25–50%), sodium dodecylbenzene sulfonate (10–25%), butanedioic acid (≤10%), sulphamic acid (≤5%), potassium hydrogen sulphate (≤5%), sodium chloride (≤5%), dipotassium peroxodisulphate (≤5%), dipotassium disulphate (≤5%), dipentene (<1%)	0.100%
**Water Acidifiers**	Agrocid Super™Oligo	Liquid	Formic acid (35–50%), propionic acid (15–30%), lactic acid (5–15%), citric acid monohydrate (1–5%), zinc chloride (0.1–1%), dicopper chloride trihydroxide (0.1–1%), sodium chloride, glucose syrup, E6-zinc = 2500 mg/kg, sorbic acid	0.050%
	Premium Acid	Liquid	Formic acid (29%), propionic acid (7.5%), lactic acid (9.2%), acetic acid (2.5%), sorbic acid (0.2%), oligofructose syrup (1%), copper sulphate pentahydrate (0.5%), sodium chloride (0.1%), zinc chelate of glycine hydrated (0.1%)	0.200%
	Ultimate acid	Liquid	Formic acid (28%), ammonium formate (7.5%), acetic acid (4.5%), propionic acid (4.5%), copper chelate of glycine hydrated (3.8%), lactic acid (3.6%), zinc chelate of glycine hydrated (3.5%), sorbic acid (0.24%), monosodium phosphate dihydrate (0.034%), sodium chloride (0.034%)	0.200%
**Water glycerides blends**	CFC Floramix	Liquid	Mono- and diglycerides of fatty acids (30%), formic acid (18%), propionic acid (9%), glycerine (7%), lactic acid (3.2%), polyethylene glycol glyceryl ricinoleate (3%), oligofructose sirup (1.5%), sorbic acid (0.3%), citric acid monohydrate (0.15%), acetic acid (0.12%)	0.150%
	FRA^®^ LAC34	Liquid	Lactic acid, glycerides of propionic and butyric acid	0.300%
	FRA^®^ Gut Balance	Liquid	Glycerides of propionic, butyric, caprylic and capric acid	0.300%

PC = Product category. ^1^: Maximum recommended dosage by the manufacturer (when animals are present). According to the instructions, in some products dose may be increased, whereas the evaluation of the optimal farm-specific dose is highly encouraged. For the products for which data were available, we included the percentage of the concentrations of individual compounds. It is important to note that all the products may contain additional proprietary ingredients used for stabilization and enhancing effectiveness.

**Table 2:** Information on the selected bacterial strains.

Species	Strain Designation	Gram Stain	Strain Type
*Campylobacter jejuni*	-	G-	wild
*Campylobacter jejuni*	-	G-	wild
*Campylobacter coli*	-	G-	wild
*Escherichia coli*	ATCC 11303	G-	reference
*Escherichia coli*	ATCC 25922	G-	reference
*Salmonella* Typhimurium	DT 120	G-	reference
*Salmonella* Typhimurium	U292	G-	reference
*Staphylococcus aureus*	DSM 102262	G+	reference
*Staphylococcus aureus*	DSM 25629	G+	reference
*Staphylococcus aureus*	-	G+	wild
*Listeria monocytogenes*	Scott A	G+	reference
*Listeria innocua*	ATCC 33090	G+	reference
*Listeria monocytogenes*	-	G+	wild

## Data Availability

None of the data presented were deposited in an official repository.
